# Change Detection: Training and Transfer

**DOI:** 10.1371/journal.pone.0067781

**Published:** 2013-06-28

**Authors:** John G. Gaspar, Mark B. Neider, Daniel J. Simons, Jason S. McCarley, Arthur F. Kramer

**Affiliations:** 1 Beckman Institute for Advanced Science and Technology and Department of Psychology, University of Illinois, Urbana-Champaign, Illinois, United States of America; 2 Department of Psychology, University of Central Florida, Orlando, Florida, United States of America; 3 Department of Psychology, Flinders University, Adelaide, South Australia; CNRS - Université Claude Bernard Lyon 1, France

## Abstract

Observers often fail to notice even dramatic changes to their environment, a phenomenon known as change blindness. If training could enhance change detection performance in general, then it might help to remedy some real-world consequences of change blindness (e.g. failing to detect hazards while driving). We examined whether adaptive training on a simple change detection task could improve the ability to detect changes in untrained tasks for young and older adults. Consistent with an effective training procedure, both young and older adults were better able to detect changes to trained objects following training. However, neither group showed differential improvement on untrained change detection tasks when compared to active control groups. Change detection training led to improvements on the trained task but did not generalize to other change detection tasks.

## Introduction

People often fail to detect changes to objects and scenes when the localizable signal produced by a change is masked or disrupted [1], [2]. Given that such “change blindness” affects performance in real-world tasks such as driving [3], improving the ability to detect changes could have practical benefits.

Evidence that change detection is amenable to learning comes from findings that expertise is associated with improved detection of domain-relevant changes. Football experts, for example, are better than non-experts at detecting changes to football images in a flicker change detection task [4]. Similarly, veterinary medicine students outperform undergraduates at detecting changes to radiograph images [5], [6]. This expert advantage disappears for images outside the observer’s area of expertise [4], [5], suggesting that experts are no better at change detection in general–they are just more familiar with the stimuli in their domain of expertise.

These group differences appear to be driven by domain-specific knowledge, implying a potential role of learning in change detection performance. They do not rule out the possibility, however, that the effects of expertise derive from more basic cognitive differences that are amenable to training.

In general, the benefits of perceptual training are specific to the trained task; training improves trained-task performance, but does not transfer to untrained tasks [7]. For example, observers trained to identify briefly presented objects showed no performance benefits for untrained objects [8]. Similar specificity of training has been shown in motion discrimination [9], orientation discrimination [10], [11], pop-out detection [12], and vernier acuity [13]. Training also tends to have limited transfer for higher-level cognitive functions including working memory [14], [15], speed of processing [16], visual search [17], [18], and multi-task performance ([19], but see [20, 21, & 22]). A recent study, however, showed that adaptive working memory training improved the change detection performance of dysphoric individuals [23], which suggests that the general ability to detect changes might be amenable to training. We might therefore expect that training to detect changes on one task might improve change detection on other tasks.

In the current work, we examined whether observers could be trained to detect changes more efficiently, and if so, whether their learning would transfer to other, untrained change detection tasks. Transfer tends to be limited when participants learn to recognize individual objects [8]. Change detection, however, imposes processing demands on working memory beyond those on object recognition. Our training task therefore employed displays with multiple objects (3 and 5), increasing the need to efficiently consolidate information into visual working memory [24], [25]. This allowed us to explore whether the ability to extract and consolidate information from multi-object displays is trainable. Furthermore, participants trained on a large number of objects of four different types. We predicted that training on a broad array of diverse objects was more likely to engender transfer to untrained stimuli.

A central component of change detection performance is the ability to encode the pre-change display, so we adaptively trained observers to encode the initial display faster while preserving their accuracy. Presumably, faster encoding should improve change detection by allowing participants to transfer pre-change information into working memory more efficiently. If improved encoding enhances change detection performance more generally, that acquired skill should transfer to untrained change detection tasks. Alternatively, if training increases familiarity with the trained objects but does not improve change detection ability, then performance gains should be limited to the trained stimuli. We tested transfer to a similar one-shot change detection task. This task was structurally similar to the training task, but the training objects were replaced with novel colored bars. We also tested transfer to a flicker change detection task with images of real-world driving scenes. The flicker paradigm was structurally dissimilar to the training task, and the images contained considerably more detail and clutter than did the arrays of objects, making the flicker task a measure of broader transfer.

Given that older adults typically demonstrate greater change blindness than younger adults [26], older adults should have more room for improvement. Consequently, older adults could benefit more than younger adults from a training regimen that focuses on improving encoding efficiency. Furthermore, our training task focused specifically on improving encoding. Age-related visual short-term memory impairments result in part from inefficient encoding [27], [28] which appears to reflect age differences in inhibitory control [29]. We included younger and older adult groups to compare age effects in training and transfer. Specifically, we predicted that increasing encoding efficiency might be more beneficial for older adults.

## Method

### Participants

40 young adults (mean age = 21.3, SD = 2.3; range = 18–28) and 40 independent-living older adults (mean age = 75.4, SD = 4.3; range = 65–84) were recruited from the Urbana-Champaign community, and were paid $10 per hour. Participants provided written consent by signing a consent form. The University of Illinois Institutional Review Board approved this procedure. All participants demonstrated normal or corrected-to-normal visual acuity (20/30 cutoff) and color vision (Ishihara Color Vision Test), and all older participants scored above 27 (out of 30) on the Mini-Mental State Exam.

### Apparatus

Five PC’s with 19-inch screens were used for the training and the flicker change detection transfer tasks. An Apple eMac with a 17-inch monitor was used for the one-shot change detection transfer task. The change detection training program was created using MATLAB® software (MathWorks™). Transfer tasks were programmed using E-prime® (Psychology Software Tools) and Vision Shell. Viewing distance for all tasks was approximately 77cm, although participants were free to move their heads.

### Training Programs

**Figure 1:**
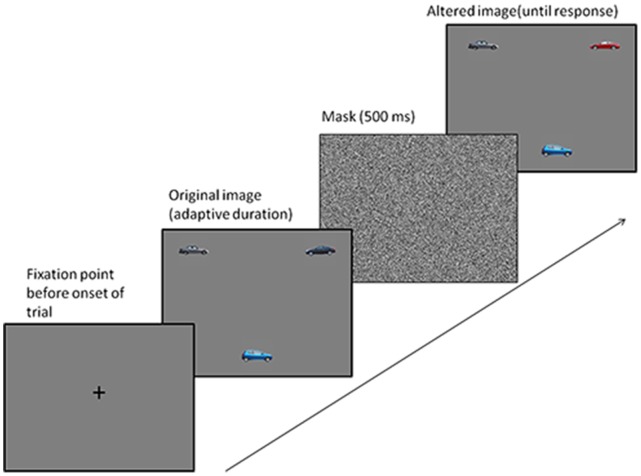
Change detection training task. Stimuli and sequence of events comprising each trial in the change detection training task.

Participants in the Change Detection training group (20 young, 20 old) practiced an adaptive change detection task (Figure 1). On each trial, participants first saw a fixation cross, followed by an original display of 3 or 5 objects, and then by a 500 ms black and white mask. Following the mask, participants saw a modified display in which one object (target) from the original image was replaced by a novel object. Participants used the mouse to select the changed object. The modified display remained visible until they responded.

The training stimuli comprised forty exemplars in each of 4 object categories (cars, signs, shapes, letters). Participants trained on one category of objects in each session. On each trial, the target object (i.e. the object that changed) was selected randomly from the 40 objects in the category, under the constraint that each object was selected as the target 10 times per session. The distracter items for each trial (i.e. those that did not change) were chosen randomly from the remaining objects. Participants trained 4 times on each object category, for a total of 16 training sessions. The order of object categories across sessions was counterbalanced between participants.

Accuracy at each set size in each session was thresholded at 75%; that is, the presentation duration (i.e. encoding time) of the original display on each trial was shortened or lengthened adaptively, using the Quest algorithm [30], to maintain 75% accuracy. The initial presentation durations for each set size were derived through pilot testing (Younger adults: set size 3 = 280 ms, set size 5 = 2012 ms; older adults: set size 3 = 413 ms, set size 5 = 2678 ms), and were the same for each session. Participants completed 200 trials of set size 3 intermixed with 200 trials of set size 5 in each hour-long training session.

Participants in the control group (20 young, 20 old) played 16 hours of computer card games (Hoyle Card Games, Encore Software, Inc. 2008).

### Transfer Tasks

#### One-shot change detection [31]

**Figure 2:**
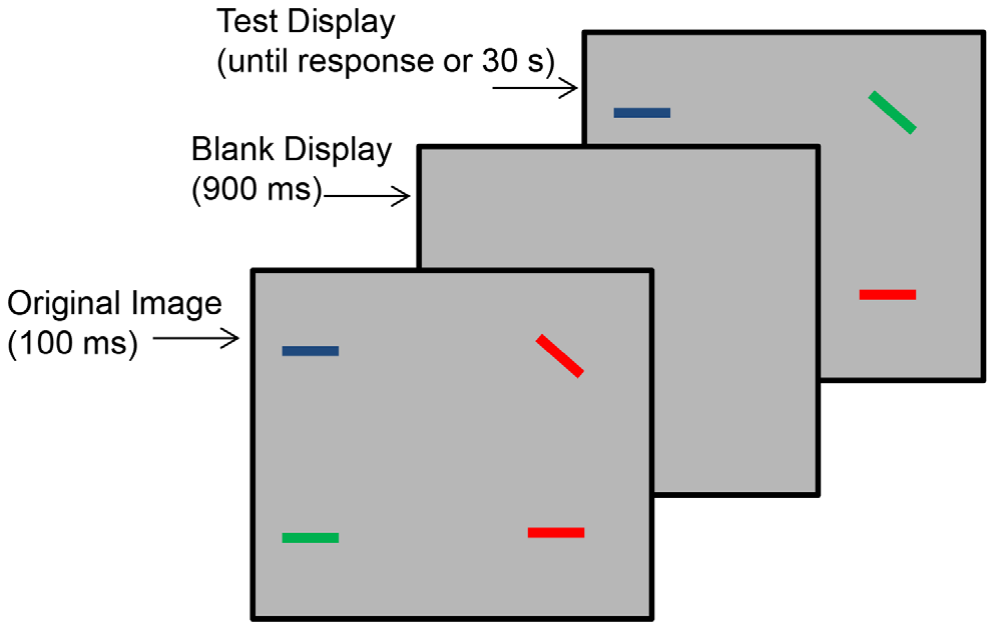
One-shot change detection task. Stimuli and sequence of events comprising one trial in the one-shot transfer task.

Participants determined whether two briefly presented displays differed (Figure 2). They first saw a briefly presented (100 ms) display containing 2, 4, or 6 colored bars that were individually randomly assigned one of four orientations (vertical, horizontal, tilted left, tilted right). That display was followed by a 900 ms blank display, which was then followed by a test display. On 50% of the trials, an item with a different color or orientation replaced one item in the original display. Participants had up to 30 seconds to indicate by key press whether the two displays were the same or different, and each trial ended when they responded. Participants completed 24 practice trials followed by 144 test trials. The location and orientation of the bars varied randomly on each trial in both the pre- and post-training sessions. This one-shot transfer task represents relatively near transfer from the training task in that this task is structurally similar to the training task, but with different timing and simpler objects.

#### Flicker change detection [26]

**Figure 3:**
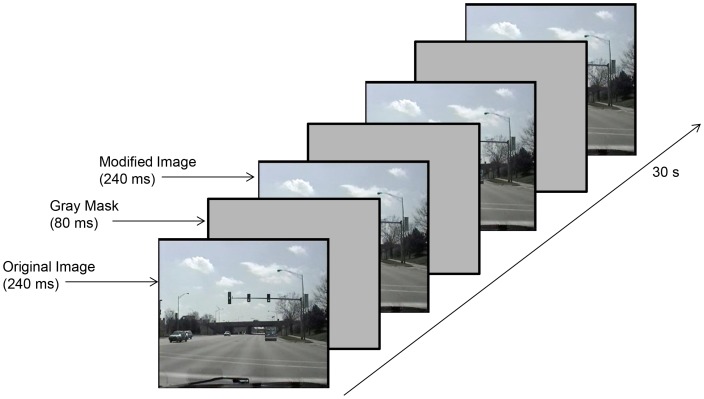
Flicker change detection task. Stimuli and sequence of events for one trial in the flicker change detection transfer task.

Participants performed a flicker change detection task with 80 pairs of photographs of driving scenes taken from the driver’s perspective (Figure 3). Each pair of images differed in one detail. Differences included color and location changes to existing objects and the removal/addition of one object (e.g. a car was present in one image and was absent in the other image). On each trial, participants saw a repeating cycle of 4 images: the original image for 240 ms, a gray mask screen (80 ms), the modified image (240 ms), and another gray mask screen (80 ms). Participants pressed a key when they detected the change. One of the two images was then presented on the screen, and the participant selected the change location with the mouse. If the participant did not respond, the trial ended after 30 s. The set of 80 image pairs was divided into two sets of 40 pairs and participants were tested on one subset prior to training and the other subset following training, with order counterbalanced across subjects. Before completing the experimental trials, participants completed one practice trial with images not from the set of 40 image pairs.

### Procedure

Following a screening session, participants completed pre-training assessments on both transfer tasks, and were then randomly assigned to either the change detection training group or the active control group. They then completed 16 one-hour training sessions, followed by a post-training transfer session. Participants completed 2–3 sessions per week and finished the study in approximately 8–9 weeks.

## Results

### Training Improvement

We assessed whether training on the adaptive change detection task improved trained-task performance by testing for improvements in encoding speed and accuracy. Analyses compared performance in the first and last session using an ANOVA with age (Young vs. Old) as a between-subject factor and session (1 vs. 16) and set size (3 vs. 5) as within-subject factors.

#### Encoding speed

**Figure 4:**
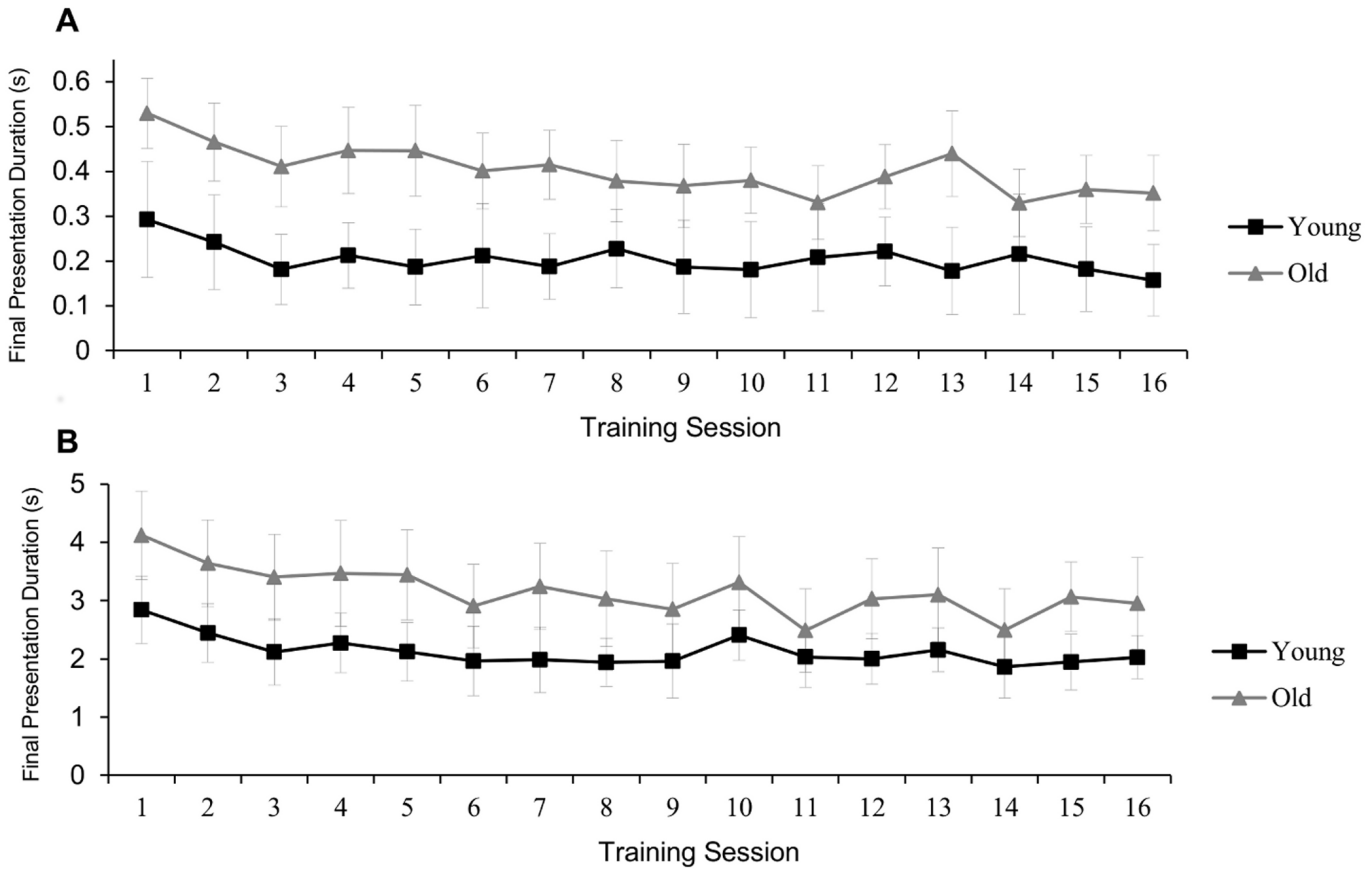
Training improvement. Final presentation duration, in milliseconds, for set size 3 (A) and 5 (B) over the course of training for the young and old change detection training groups. Error bars represent 95% within-subjects confidence intervals [37], [38].

During training, the display duration was adjusted dynamically to maintain 75% accuracy. Shorter display durations indicate faster encoding of the initial display. Thus, we used the presentation duration of the final successful trials of set size 3 and 5 within each session as a measure of the speed of encoding (see Figure 4). Overall, participants were faster at set size 3 than at set size 5 [F(1, 38) = 37.2, p<.001, η^2^_p_ = .50]. Young adults achieved shorter presentation durations than did older adults at both set size 3 [F(1, 38) = 37.2, p<.001, η^2^_p_ = .50] and set size 5 [F(1, 38) = 8.3, p = .006, η^2^_p_ = .18]. To analyze performance over the course of training, we compared final presentation durations from the first training session (session 1) and the last training session (session 16). With practice, the duration should decrease, indicating faster encoding of the initial display. Participants improved over the course of training, achieving shorter presentation durations in session 16 than in session 1 for both set size 3 [F(1, 38) = 17.8, p<.001, η^2^_p_ = .32] and set size 5 [F(1, 38) = 12.8, p = .001, η^2^_p_ = .25]. Younger and older adults showed similar reductions in encoding time for set size 3 (15% for younger adults and 23% for older adults; [F(1, 38) = .29, p = .60, η^2^_p_ = .01]) and for set size 5 (9% for younger adults and 5% for older adults; [F(1, 38) = .62, p = .53, η^2^_p_ = .01]). Both age groups improved more for set size 3 than for set size 5, [F(1, 38) = 10.93, p = .002, η^2^_p_ = .22], likely as a result of the choice of initial presentation duration for each set size, which may have modulated the room for improvement at each set size. Neither the age by session interaction, [F(1, 38) = .39, p = .52, η^2^_p_ = .01], nor the interaction between age, session, and set size, [F(1, 38) = .39, p = .54, η^2^_p_ = .01], was significant.

#### Accuracy

Although we used the Quest algorithm to select presentations durations that would maintain 75% accuracy, participants achieved slightly higher accuracies due to the limited number of trials in each session. Accuracy improved between session 1 (80.3%) and session 16 (83.5%), [F(1, 38) = 9.78, p = .003, η^2^_p_ = .21], and this improvement was greater for set size 5 than for set size 3, [F(1, 38) = 6.73, p = .013, η^2^_p_ = .15]. The main effect of age, [F(1, 38) = .38, p = .54, η^2^_p_ = .01], and age by session interaction, [F(1, 38) = 1.83, p = .18, η^2^_p_ = .05], were not significant. However, older adults did improve more than younger adults at set size 5 [F(1, 38) = 4.58, p = .04, η^2^_p_ = .11].

### Transfer Task Performance

If training on a change detection task improves change detection performance in general, we should expect transfer from the trained task to other change detection tasks. The One-Shot Change Detection transfer task is structurally similar to our training task, so if we successfully trained the underlying process of change detection, we should be most likely to see transfer to that task. The Flicker Change Detection task uses richer displays and an ongoing search for changes, so it constituted a test of somewhat broader transfer. In addition to the traditional hypothesis tests, we also report p_BIC_(H_1_|D) [32], [33] for the interactions gauging transfer of training. This statistic provides an estimate of the posterior probability of the alternative hypothesis given the observed data, allowing conclusions either against or in favor of the null hypothesis. A value of p_BIC_(H_1_|D) less than.5 favors the null hypothesis of no transfer, and a value greater than.5 favors the alternative of transfer.

#### One-shot change detection

**Figure 5:**
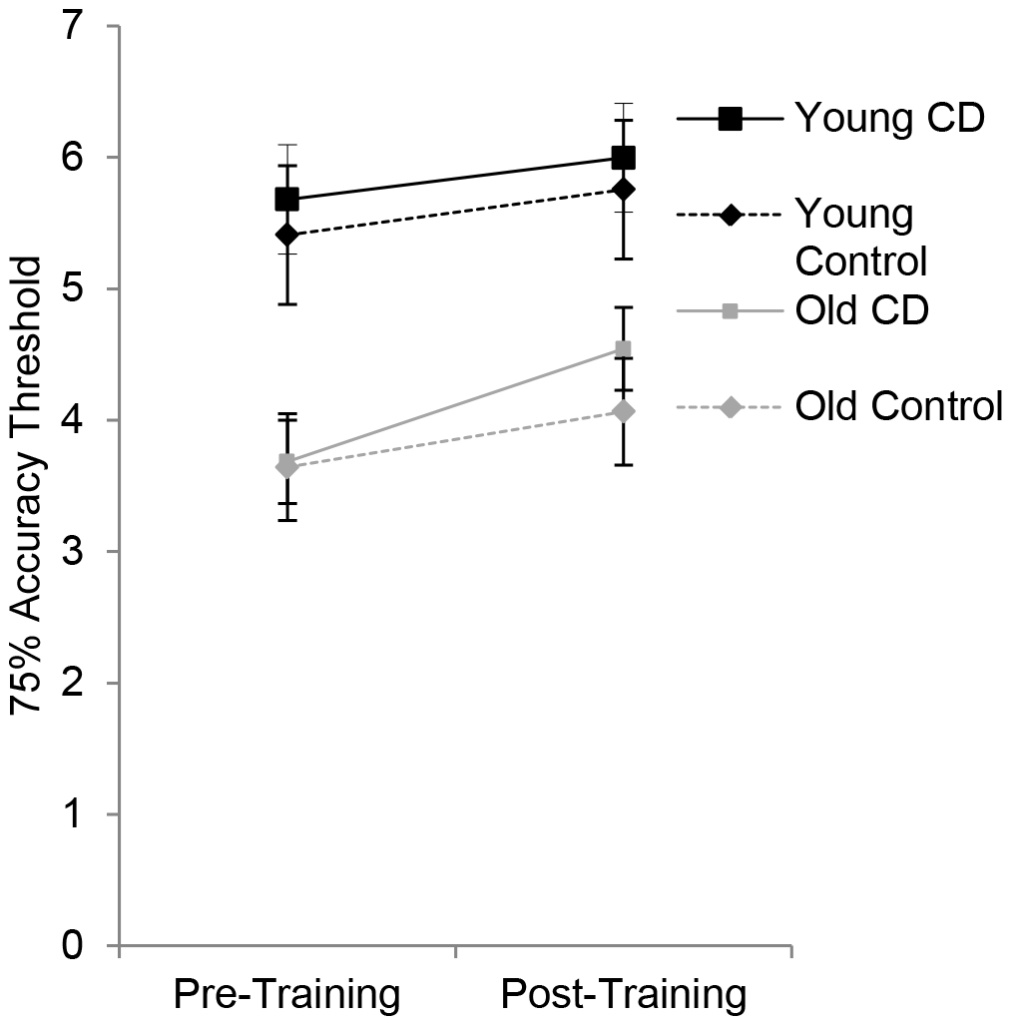
One-shot change detection transfer task. 75% accuracy thresholds on the one-shot change detection task, for each group pre- and post-training. CD refers to the change detection training groups. Error bars represent 95% within-subjects confidence intervals [37], [38].

In the One-Shot task, the percent correct for each set size was used to derive 75% accuracy thresholds (i.e. the estimated set size required to yield 75% accuracy) for each group at the pre- and post-training testing sessions (Figure 5). These results were entered into an ANOVA with session as a within-subjects factor and training group (training vs. control) and age (young vs. old) as between-subjects factors. Thresholds increased with practice, [F(1, 76) = 15.6, p<.001, η^2^_p_ = .17], but, importantly, the training group did not show larger threshold improvement than did the control group, [F(1, 76) = .69, p = .69, η^2^_p_ = .009, p_BIC_(H_1_|D) = .16]. An analysis of statistical power revealed that the probability of detecting a small, medium and large effect size (η^2^ = .01,.06,.14) for the group by session interaction was.48,.99, and.99, respectively. The estimated Bayesian posterior probability for the group by session interaction was low, suggesting that the data accord better with the null hypothesis. Averaging across sessions, younger adults had larger thresholds than did older adults, [F(1, 76) = 43.7, p<.001, η^2^_p_ = .37].

To examine whether participants who improved more during training showed greater transfer to the One-Shot task, we performed a median split based on percent reduction in final training duration (separately for each age group) and ran an ANOVA with training improvement (high vs. low) as a between-subjects factor and session as a within-subjects factor. Training improvement did not interact significantly with One-Shot task improvement, [F(1, 38) = .90, p = .35, η^2^_p_ = .02], indicating that greater improvements during training did not lead to greater transfer of training.

#### Flicker change detection task

**Figure 6:**
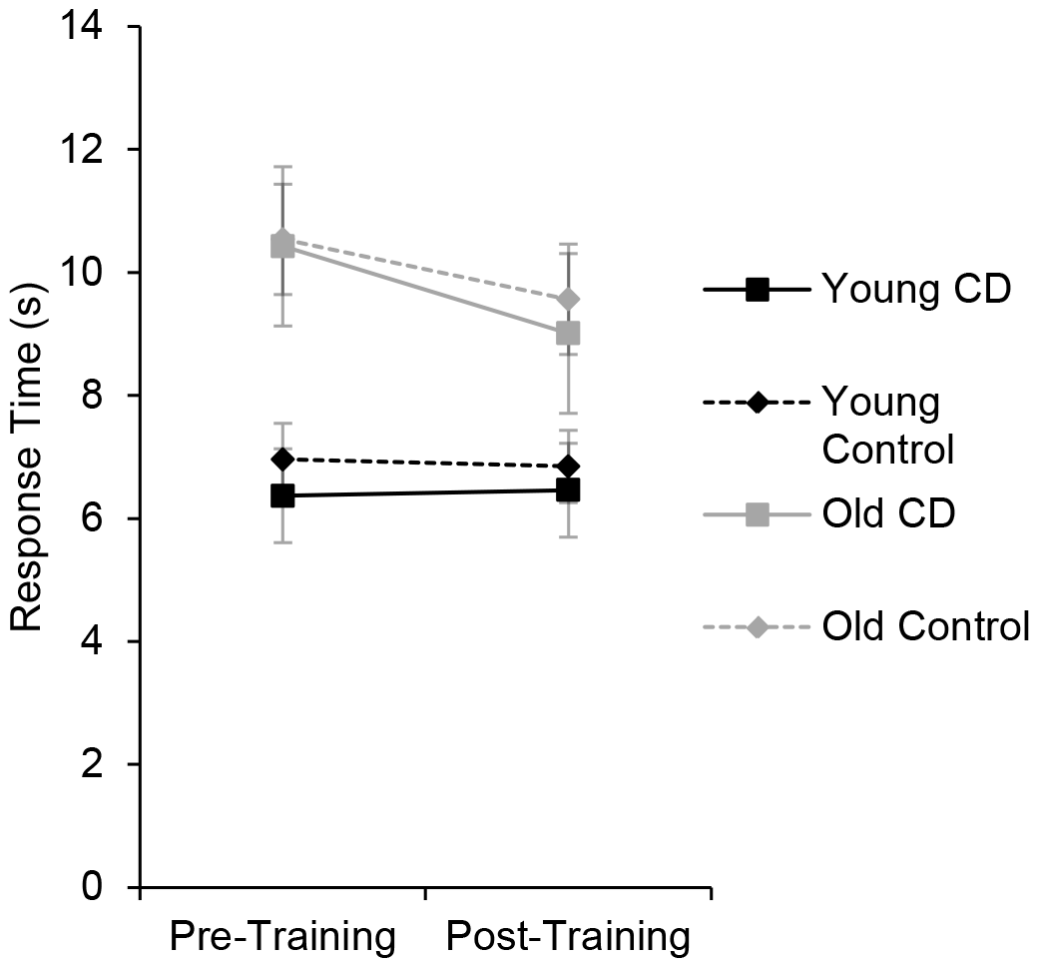
Flicker change detection transfer task. Response time, in seconds, to detect changes on the flicker change detection task for each group pre- and post-training. CD refers to the change detection training groups. Error bars represent 95% within-subjects confidence intervals [37], [38].

Change detection performance was defined as the time to accurately detect the change in each display (Figure 6). Time-outs and incorrect responses were excluded from analysis. For all analyses, we ran ANOVAs with session (Pre- vs. Post-training) as a within-subjects factor and age (Young vs. Old) and training group (training vs. control) as between-subjects factors. Participants were faster in the post-training session (7.7 s) than in the pre-training session (8.4 s), [F(1, 76) = 5.1, p = .03, η^2^_p_ = .06]. Critically, there was no additional benefit of change detection training, indicated by the lack of a group by session interaction [F(1, 76) = .05, p = .83, η^2^_p_ = .001, p_BIC_(H_1_|D) = .14]. An analysis of statistical power revealed that the probability of detecting a small, medium and large effect size (η^2^ = .01,.06,.14) for the group by session interaction was.22,.98, and.99, respectively. The estimated Bayesian posterior probability for the group by session interaction was low, suggesting that the data were better fit by the null hypothesis. Change detection training thus did not transfer to another change detection task that used richer displays and an ongoing search for changes; the faster performance on the post-test likely resulted from practice with the flicker task during the pre-test.

Overall, young adults (6.4 s) were faster to detect changes than were older adults (9.7 s), [F(1, 76) = 119.8, p<.001, η^2^_p_ = .61]. Older adults improved more on the Flicker Change Detection task than did young adults, as indicated by a significant age by session interaction [F(1, 76) = 4.9, p = .03, η^2^_p_ = .06].

We also examined whether the participants who showed greater training improvement were more likely to display transfer, using separate ANOVA for each age group with training improvement (high vs. low) as a between-subjects factor and session as a within-subjects factor. Training improvement (high vs. low), did not interact significantly with Flicker Change Detection improvement, [F(1, 38) = 1.53, p = .22, η^2^_p_ = .04], indicating that improvement in the training task did not predict transfer.

## Discussion

Although training on a change detection task improved performance, with participants requiring less encoding time for accurate change detection, that improvement did not transfer to a structurally similar one-shot change detection task or to a flicker change detection task. Both the training group and the control group improved when they completed the transfer tasks a second time, but they improved to equal extents. Change detection training did not improve change detection on other tasks, either in a similar one-shot change detection task with different stimuli or in a flicker change detection task with real-world images.

Given the lack of differential performance for the training group and the control group, performance improvements on the transfer tasks presumably resulted from practice on those tasks rather than transfer of training. This lack of transfer is consistent with the perceptual training literature in which training often improves trained-task performance, but transfer tends to be limited and narrow [11], [13], [16]. The one-shot change detection transfer task is structurally the same as our training task, differing only in timing and the use of simpler, novel objects (colored bars instead of cars, signs, etc.). The lack of transfer from our object change detection task to another one-shot change detection task suggests that training effects were limited to the trained objects and did not enhance the underlying change detection processes or other mechanisms and strategies that would aid performance of the same task with different objects. That is, the improvements during training (i.e. reductions in encoding time) likely resulted from increased familiarity with the trained stimuli rather than from an improvement in underlying change detection ability.

Though the trained stimulus set was relatively large, observers can differentiate between studied and altered objects, even when asked to remember hundreds of objects [34], [35]. In the present study, training improvements resulted from increased familiarity with the trained objects. Importantly, within the parameters of the training task, much of the improvement in encoding time occurred within the first three sessions (Figure 4), suggesting that participants became familiar with the objects in relatively little time (∼3–4 hours). Increased familiarity with the trained stimuli would not, however, improve the ability to detect changes in any untrained stimuli, such as those used in the one-shot change detection transfer task. Our results suggest that training would be similarly specific for other sets of objects (e.g. colored bars, detailed pictures). This stimulus specificity is consistent with evidence that expert observers fail to outperform non-experts when the images are unrelated to their domain of expertise [4], [5]. Our change detection training participants likely became experts in rapidly encoding the trained stimuli, not necessarily in detecting changes. However, it is unknown whether training would transfer to untrained configurations of the same objects (e.g. rotated trained objects).

One caveat to our evidence for limited transfer comes from the nature of our training procedure. We adaptively manipulated encoding time, but several other processes (e.g. memory, comparison) contribute to successful change detection [36], and training those processes might lead to broader improvements in change detection ability. While it is likely that improved encoding was primarily responsible for improvements on the training task, we were unable to assess whether changes in other processes involved in change detection contributed to improvement in the training task. Future research could adaptively adjust other components of the change detection task (e.g. the blank duration) to see whether training those aspects of performance would lead to broader transfer.
